# Compounds from *Cyclocarya paliurus* leaves inhibit binary division of methicillin-resistant *Staphylococcus aureus* by disrupting FtsZ dynamic

**DOI:** 10.3389/fmicb.2025.1622623

**Published:** 2025-06-18

**Authors:** Wenlong Chen, Shuixian Zhang, Chunxu Huang, Zhiming Hu, Ting Cao, Jun Mou, Xinxia Gu, Meiling Sun, Jie Liu

**Affiliations:** ^1^Center for Infectious Disease and Vaccine Research, West China Hospital, West China School of Medicine, Sichuan University, Chengdu, China; ^2^Department of Healthcare Intelligence, University of North America, Fairfax, VA, United States

**Keywords:** antimicrobial resistance, filamenting temperature-sensitive mutant Z, methicillin-resistant Staphylococcus aureus, FtsZ dynamics, cell division

## Abstract

The escalating threat of methicillin-resistant *Staphylococcus aureus* (MRSA) necessitates novel therapeutic strategies. Our previous work suggested that an extract from *Cyclocarya paliurus* leaves (ECPL) inhibits MRSA by targeting the cell division protein FtsZ. Here, guided by anti-MRSA activity, we isolated three compounds from ECPL: asiatic acid (AA), maslinic acid (MA), and ursolic acid (UA). They exhibited antibacterial activity against MRSA and induced cell elongation, indicative of division arrest. Time-kill assays showed AA and MA are bactericides, while UA is bacteriostatic. Mechanistically, these compounds disrupt cell division by differentially affecting FtsZ dynamics: AA promotes polymerization, whereas MA and UA inhibit it. SPR analysis showed direct FtsZ binding to AA (Kd = 2.4 μM), MA (Kd = 9.8 μM), and UA (Kd = 0.7 μM). Molecular docking predicted a shared FtsZ binding pocket but revealed that AA adopts a distinct conformation driven by unique interactions, including a hydrogen bond with Arg191—an interaction not observed for MA or UA, which instead form hydrogen bonds with Thr265 and Thr309. Despite these divergent effects on polymerization and distinct binding modes, all compounds ultimately disrupted Z-ring assembly and septum formation. In a murine skin infection model, AA, selected for its bactericidal activity and unique FtsZ modulation mechanism, significantly reduced bacterial burden and accelerated wound healing. Collectively, our findings validate these compounds as direct FtsZ-targeting agents and establish AA as a promising anti-MRSA lead compound with a novel mechanism disrupting the bacterial divisome.

## 1 Introduction

The rise of antimicrobial resistance (AMR) poses one of the most pressing challenges to public health and modern healthcare systems (Abbas et al., 2024). In 2019, approximately 4.95 million deaths were linked to bacterial AMR, with 1.27 million directly attributed to it. The six leading pathogens responsible for these deaths include *Escherichia coli*, *Staphylococcus aureus*, *Klebsiella pneumoniae*, *Streptococcus pneumoniae*, *Acinetobacter baumannii*, and *Pseudomonas aeruginosa* (Miller and Arias, 2024). Among them, methicillin-resistant *Staphylococcus aureus* (MRSA) is a major pathogen whose lethality is heightened by AMR, causing a significant number of invasive infections and deaths worldwide (Dance, 2024). MRSA remains a leading cause of mortality associated with AMR across various regions globally (Larsen et al., 2022).

Addressing the AMR crisis is significantly hampered by the stagnation in the development of novel antimicrobial agents (Dance, 2024). While a few new agents have been approval for specific indications (Upadhayay et al., 2023), they have not kept pace with the rapid emergence of AMR. Alarmingly, analyses of recently approved antibiotics and those in clinical development over the past decade reveal that over three-quarters are structural derivatives of existing drug classes or target established mechanisms (Zhao et al., 2024). This reliance on familiar scaffolds and targets limits their long-term efficacy against continuously adapting pathogens, highlighting the critical need for antimicrobial agents with fundamentally novel chemical structures and distinct mechanisms of action.

Targeting essential bacterial processes like cell division offers a promising strategy for developing such novel antibiotics (den Blaauwen et al., 2014). The bacterial tubulin homolog Filamenting temperature-sensitive mutant Z (FtsZ) plays a central and indispensable role in initiating cell division by forming the dynamic Z-ring, a critical scaffold for the divisome complex responsible for septum synthesis (Bisson-Filho et al., 2017; McQuillen and Xiao, 2020). Importantly, FtsZ is highly conserved across diverse bacterial species yet lacks a close homolog in humans, making it an attractive target for antibacterial agents with potentially low off-target toxicity (Li and Ma, 2015). Consequently, disrupting FtsZ assembly or function presents a compelling strategy to block bacterial replication. Considerable interest has led to the identification of FtsZ inhibitors like PC190723, which validated FtsZ as a druggable target against Gram-positive pathogens (Haydon et al., 2008). However, its clinical progression was limited by suboptimal pharmacokinetics and inadequate *in vivo* efficacy. Subsequent inhibitors, spanning diverse chemical scaffolds including natural products, have been reported but often suffer from limited potency or lack of demonstrated efficacy in animal models, underscoring the urgent need for more effective FtsZ inhibitors for clinical application (Wang et al., 2024).

Natural products, with their vast chemical diversity, remain a historically rich and currently vital source for novel drug leads (Dai et al., 2020). Consistent with this, our previous work identified that an extract from *Cyclocarya paliurus* leaves (ECPL), a medicinal plant, possesses anti-MRSA activity by impairing FtsZ function (Sun et al., 2023). However, the specific bioactive components within ECPL responsible for FtsZ modulation, their precise molecular interactions with FtsZ remained undetermined.

Here, addressing these gaps, we aimed to isolate and chemically identify the FtsZ-active constituents from ECPL. Utilizing a comprehensive approach combining antibacterial activity-guided fractionation, structural elucidation, biochemical, biophysical, cellular imaging, and *in vivo* efficacy studies, we sought to systematically elucidate the mechanisms underlying their anti-MRSA activity, define their precise molecular interactions with FtsZ, and evaluate their potential as novel therapeutic candidates. Through this integrated investigation, we aimed to gain detailed molecular insights into the intricate interaction between these compounds and FtsZ dynamics, ultimately contributing valuable knowledge to the development of next-generation antibacterial agents targeting the bacterial divisome.

## 2 Materials and methods

### 2.1 Bacterial strains and reagents

*Staphylococcus aureus* ATCC25923 and *Escherichia coli* ATCC25922 were obtained from the American Type Culture Collection (ATCC, Manassas, VA, USA). The Methicillin-resistant *S. aureus* (MRSA) clinical isolate MRSA-4, previously confirmed as *mecA*-positive and characterized for its antibiotic resistance profile (Sun et al., 2023), was used in this study. *Staphylococcus epidermidis* (GDMCC 1.143), *Enterococcus faecalis* (GDMCC 1.164), and *Salmonella enterica* subsp. *enterica* (GDMCC 1.1114) were obtained from the Guangdong Microbial Culture Collection Center (GDMCC, Guangzhou, China). The *Pseudomonas aeruginosa* PA14 strain was kindly provided by Professor Min Wu (Southwest Medical University, China). All bacterial strains were routinely cultured in Mueller-Hinton broth (MHB) or Luria-Bertani (LB) broth medium at 37°C with shaking at 200 rpm. Vancomycin was purchased from Solarbio Science & Technology Co., Ltd. (Beijing, China), and oxacillin was obtained from Taiji Group Hengsheng Pharmaceutical Co., Ltd. (Chengdu, China). The polyclonal rabbit primary anti-body to FtsZ was purchased from Abcam PLC (Cambridge, UK). Alexa488-conjugated goat anti-rabbit IgG was purchased from ZSGB-BIO Biotechnology (Beijing, China). DAPI staining solution was purchased from Beyotime Biotechnology (Shanghai, China).

### 2.2 Minimum inhibitory concentration determination

The minimum inhibitory concentrations (MICs) of the test compounds (AA, MA, and UA) were determined using the standard broth microdilution method in accordance with the Clinical and Laboratory Standards Institute (CLSI) guidelines (Humphries et al., 2021). Bacteria were cultured in MHB or LB broth at 37°C with shaking (200 rpm) to the logarithmic phase, then diluted to a final density of 1 × 10^6^ CFU/mL. Test compounds (dissolved in DMSO) and positive controls (vancomycin and oxacillin) were diluted in MHB and serially twofold diluted in 96-well plates. Each well was inoculated with 100 μL of bacterial suspension (1 × 10^6^ CFU/mL), yielding a final bacterial density of 5 × 10^5^ CFU/mL and a total volume of 200 μL. The final concentrations tested were 128 to 1 μg/mL for AA, MA, and UA; 16 to 0.5 μg/mL for vancomycin; and 64 to 0.0625 μg/mL for oxacillin. DMSO was used as a solvent control. Plates were incubated statically at 37°C for 18–24 h. After incubation, the OD_600_ was measured using a microplate reader (SpectraMax i3x, Molecular Devices, CA, USA). The MIC was defined as the lowest compound concentration that caused ≥ 90% inhibition of bacterial growth compared to the drug-free growth control. All tests were performed in three independent biological replicates.

### 2.3 Isolation of compounds

Dried *Cyclocarya paliurus* leaves were ground and passed through a 40-mesh sieve. A total of 10 kg of powdered material was extracted twice with 100 L of water at 90°C for 2 h each time. The combined supernatants were concentrated under reduced pressure to obtain 2 kg of crude extract (ECPL).

ECPL (2 kg) was dissolved in water and loaded onto a D101 resin column (25 kg). Stepwise elution was performed with ddH_2_O followed by ethanol gradients (20, 40, 60, 80, and 100%), with each step comprising 10 bed volumes (BV) at a flow rate of 5 BV/h. The 60 and 80% ethanol fractions, which exhibited the strongest anti-MRSA-4 activity based on MIC evaluation, were combined to yield ~80 g of enriched extract.

This extract was dissolved in dichloromethane and fractionated on a polyamide column (Aladdin, China; 2 kg) using a stepwise DCM–MeOH gradient (v/v: 10:0, 8:2, 2:1, 5:5, 1:2, 0:10). Each step involved 10 BV at a flow rate of 5 BV/h. The 8:2 DCM–MeOH fraction (~40 g) showed the highest anti-MRSA-4 activity.

The ~40 g active fraction was dry-loaded onto a silica gel column (800 g, 100–200 mesh) after pre-adsorption onto 60 g of silica gel. Separation was performed using a Isolera One medium-pressure preparative chromatography system (Biotage, Sweden) with a DCM–MeOH gradient (v/v: 10:0, 9:1, 8:2, 7:3, 5:5), each step comprising 10 BV. Fractions were monitored by UV at 360 nm. The 9:1 DCM–MeOH fraction (~10 g) demonstrated the most potent anti-MRSA-4 activity.

Further purification was conducted on the 9:1 DCM–MeOH fraction using a second silica gel column (40 g, pre-adsorbed onto 40 g of silica gel), eluted with a stepwise petroleum ether–ethyl acetate gradient (v/v: 10:0, 9:1, 8:2, 7:3, 6:4, 5:5, 0:10; ~10 BV per step). The most active sub-fraction (~2 g) was obtained with 100% ethyl acetate.

Final purification was achieved by repeated preparative reversed-phase HPLC using an ÄKTA Avant 150 liquid chromatography system (GE Healthcare, USA) equipped with a C18 column (Wuhan Ruihe Chromatography Technology Co., Ltd., Wuhan, China; 20 × 250 mm, 10 μm). A linear gradient of 5–95% methanol in water over 20 min was used at a flow rate of 2 mL/min, with UV detection at 230 nm. This process yielded compounds 1, 2, and 3. Purity (>95%) was confirmed using an Agilent 1,260 analytical HPLC system (Agilent Technologies, USA) under identical gradient and detection conditions.

### 2.4 Identification of compounds

Structural elucidation of compounds **1**–**3** was accomplished by comprehensive spectroscopic analyses, including nuclear magnetic resonance spectra (^1^H NMR and ^13^C NMR) and high-resolution mass spectrometry (HRMS). ^1^H NMR (400 MHz) and ^13^C NMR (100 MHz) spectra were recorded using a Bruker AVANCE NEO 400 MHz spectrometer in DMSO-d6. HRMS data were acquired using a Thermo Scientific Q Exactive Plus spectrometer with electrospray ionization (ESI) in negative mode. The chemical structures of the isolated compounds were confirmed by comparison of their NMR and HRMS data with those reported in the literature.

### 2.5 Time-dependent killing assays

An overnight culture of MRSA-4 was diluted 1:100 in fresh MHB and incubated at 37°C with shaking (200 rpm) until reaching mid-log phase (OD600 = 0.4–0.6). The culture was then adjusted to 1 × 10^6^ CFU/mL and treated with AA, MA, or UA at concentrations of 1×, 2×, and 4 × MIC. 2 × MIC of vancomycin was used as a positive control, and an equivalent volume of DMSO served as the negative control. The cultures were incubated at 37°C with shaking (200 rpm) during the assay. At designated time points (0, 1, 2, 4, 8, and 24 h), samples were collected, serially diluted tenfold in sterile PBS, and 100 μL of appropriate dilutions were plated on LB agar. Plates were incubated at 37°C for 18–24 h, and viable bacteria were quantified. All experiments were performed in biological triplicates.

### 2.6 Scanning electron microscopy analysis

Scanning electron microscopy (SEM) was used to observe the morphological changes in bacterial cells treated with the isolated compounds. *S. aureus* ATCC 25923 and MRSA-4 were grown in MHB to mid-log phase (OD600 = 0.4–0.6) as previously described. The bacteria cultures were treated with AA, MA, or UA at 2 × MIC for 2 h at 37°C with shaking at 150 rpm. Untreated cultures (PBS group) served as the growth control. After incubation, bacterial cells were harvested by centrifugation and washed three times with PBS (pH 7.2). Coverslips (8 mm diameter) were cleaned with absolute ethanol under ultrasonication, air-dried, immersed in 0.1% (w/v) chitosan solution (dissolved in 1% acetic acid) for 5 s, and air-dried again at room temperature (Su et al., 2022). A 25 μL aliquot of the washed bacterial suspension was added to each coverslip and allowed to adhere for 1 h at room temperature. Adhered cells were fixed with 1 mL of 2.5% (v/v) glutaraldehyde at 4°C overnight. Fixed samples were rinsed 2–3 times with PBS (5 min per rinse), then dehydrated through a graded ethanol series: 50, 70, and 90% ethanol (each once for 5 min), followed by 100% ethanol (three times for 5 min each). After dehydration, samples were air-dried overnight at room temperature. SEM imaging was performed using an EVO 10 scanning electron microscope (Zeiss, Jena, Germany). Cell long-axis statistical analysis was conducted using ImageJ software (Version 1.42q, National Institutes of Health, MD, USA).

### 2.7 Transmission electron microscopy analysis

MRSA-4 was cultured in MHB to mid-log phase as described above. The cultures were then treated with AA, MA, or UA (at 1 × MIC) or with an equal volume of PBS (negative control) for 4 h at 37°C. After treatment, bacterial suspensions were collected by centrifugation at 13,000 × g for 3 min, and the resulting pellets were washed with PBS. Pellets were fixed in 2.5% glutaraldehyde prepared in 0.1 M cacodylate buffer (pH 7.2). Following fixation, samples were dehydrated in a graded ethanol series (30 to 100%), with 100% ethanol replaced three times. Samples were infiltrated with acetone/Epon-812 resin mixtures at ratios of 3:1, 1:1, and 1:3, then embedded in pure Epon-812 resin. Ultrathin sections (60–90 nm) were cut and placed on copper grids. The sections were stained with saturated uranyl acetate for 10–15 min, followed by lead citrate for 1–2 min. Structural and morphological changes in the bacterial cells were observed using a JEOL JEM-1400FLASH transmission electron microscope (JEOL, Japan).

### 2.8 Structured illumination microscopy analysis

MRSA-4 was cultured in MHB at 37°C to mid-logarithmic phase and subsequently treated with AA, MA, or UA at concentration of 1 × MIC, or with PBS as a control, for 4 h at 37°C. Following treatment, cells were harvested by centrifugation, washed once with PBS, and fixed with 4% (w/v) paraformaldehyde in PBS for 15 min at room temperature. Fixed cells were then washed twice with PBS. The pellets were resuspended in 90 μL GTE buffer (50 mM glucose, 25 mM Tris–HCl, pH 8.0, 10 mM EDTA) containing 10 μL lysozyme (3 mg/mL) and incubated for 3 min at room temperature. The suspension was applied onto poly-L-lysine-coated glass slides and allowed to adhere. After removing excess liquid, the slides were blocked with PBS containing 2% (w/v) BSA and 0.1% (v/v) Tween-20 for 15 min. Slides were incubated overnight at 4°C with polyclonal rabbit anti-FtsZ primary antibody, followed by three washes with PBST (PBS containing 0.1% Tween-20). Alexa Fluor 488-conjugated goat anti-rabbit IgG as the secondary antibody was then applied and incubated for 1 h at room temperature in the dark. After washing, nuclei were stained with DAPI for 3–5 min, followed by 2–3 times PBS washes to remove excess stain. Coverslips were mounted with 50% (v/v) glycerol in PBS and sealed with nail polish. Z-ring structures were visualized using structured illumination microscopy (N-SIM S, Nikon, Tokyo, Japan).

### 2.9 FtsZ polymerization assay

To assess the effects of AA, MA, and UA on FtsZ polymerization, an *in vitro* FtsZ polymerization assay was performed. Recombinant SaFtsZ (FTZ02) proteins were purchased from Cytoskeleton, Inc. SaFtsZ (10 μM) was dissolved in 50 mM PIPES buffer (pH 6.8) and pre-incubated with varying concentrations of test compounds (0, 0.25 × MIC, 0.5 × MIC) or 1% DMSO at 25°C for 20 min. Polymerization was initiated by the addition of polymerization buffer components, bringing the final concentrations to 250 mM KCl and 5 mM MgCl_2_ Absorbance at 360 nm (A_360_) was monitored for 5 min to establish a stable baseline. GTP was then added to a final concentration of 1 mM. Polymerization was tracked by measuring A_360_ for 30 min using a microplate reader (SpectraMax i3x, Molecular Devices, CA, USA).

### 2.10 Surface plasmon resonance analysis

SPR analysis was performed using a Biacore X100 system (Cytiva, USA) equipped with a CM5 sensor chip at 25°C. SaFtsZ protein was immobilized onto the chip surface via standard amine coupling. Briefly, flow cells 1 (Fc1) and 2 (Fc2) were activated with a 1:1 mixture of 0.4 M EDC and 0.1 M NHS for 7 min. The SaFtsZ protein was diluted to 40 μg/mL in 0.1 M sodium acetate buffer (pH 4.0) and immobilized onto Fc2. The response units (RU) reaching an immobilization level of approximately 4693.2. Unreacted sites in both flow cells were blocked with ethanolamine for 7 min. Subsequently, analyte solutions containing test compounds at varying concentrations were injected over both flow cells, and binding responses were monitored in real time. Data analysis was performed using Biacore Evaluation Software v2.0.2.

### 2.11 Molecular docking

Molecular docking was performed to investigate the potential binding mode and interactions of AA, MA, and UA with SaFtsZ. The 2D chemical structures of the three compounds were retrieved from the PubChem database, and their initial 3D conformers were generated using Chem3D. Energy minimization was carried out using the MM2 force field to obtain stable conformations. The minimized ligand structures were then prepared for docking using AutoDock Tools, including the addition of hydrogen atoms and conversion to PDBQT format. The crystal structure of FtsZ (PDB ID: 6KVP) (Ferrer- González et al., 2019) was downloaded from the Protein Data Bank (Burley et al., 2022). Protein preparation involved removing co-crystallized ligands and water molecules using PyMOL, followed by further processing in AutoDock Tools where polar hydrogen atoms were added and Gasteiger partial charges were assigned. The prepared protein structure was saved in PDBQT format for docking calculations. Docking calculations were conducted using AutoDock Vina (Eberhardt et al., 2021). Structural visualization, analysis of ligand–protein interactions, and figure generation were carried out using UCSF ChimeraX (Meng et al., 2023).

### 2.12 MRSA skin infection model

All animal experiments were conducted in accordance with the NIH Guide for the Care and Use of Laboratory Animals and the institutional guidelines of West China Hospital, Sichuan University. The study was approved by the Institutional Animal Care and Use Committee (HX20220224085). Female BALB/c mice (6–8 weeks old) were obtained from Chengdu DOSSY Experimental Animal Co., Ltd. (Chengdu, China) and randomly assigned to five groups (*n* = 6 per group). On day 0, mice were subcutaneously inoculated with 2 × 10^8^ CFUs of MRSA-4 suspended in 50 μL of sterile PBS. Treatment commenced 24 h post-infection. Mice received once daily subcutaneous injections at the site of infection for four consecutive days with either AA (0.2, 0.4, or 0.8 mg/kg), vancomycin (0.8 mg/kg, positive control), or PBS (negative control). Lesion progression was monitored daily by photographing the infected area, and wound sizes were quantified using ImageJ software (Version 1.42q, NIH, USA). On day 5 post-infection, mice were anesthetized and euthanized. The whole lesioned skin tissues were aseptically excised, homogenized in 1 mL of sterile PBS, and serially diluted. Aliquots were plated on LB agar and incubated at 37°C overnight to determine bacterial burdens.

### 2.13 Statistical analysis

Statistical analyses were performed using GraphPad Prism 9.5 software (GraphPad Software Inc., San Diego, CA, USA). The specific statistical methods used for each experiment are detailed in the figure legends. Differences with *p* values less than 0.05 were considered statistically significant.

## 3 Results

### 3.1 Isolation and identification of anti-MRSA compounds from ECPL

To identify the active components responsible for the anti-MRSA activity of ECPL, we performed antibacterial activity-guided fractionation. This process, involving successive chromatographic separations monitored by MIC assays against MRSA-4, was performed as detailed in the Methods section. Through this approach, three triterpenoid compounds (1–3) exhibiting anti-MRSA-4 activity were isolated from the active fractions. Their structures were subsequently determined by comprehensive spectroscopic analysis, primarily using HRMS and NMR.

Compound 1 exhibited a molecular ion peak at *m/z* 487.3432 [M − H]^−^, consistent with the molecular formula C_30_H_48_O_5_. The ^1^H NMR spectrum showed characteristic signals for six methyl groups [*δ* 1.05 (s, 3H), 0.92 (d, *J* = 5.7 Hz, 6H), 0.82 (d, *J* = 6.3 Hz, 3H), 0.74 (s, 3H), 0.54 (s, 3H)], an olefinic proton (*δ* 5.13, d, *J* = 3.7 Hz, 1H), and a carboxylic acid proton (*δ* 11.93, s, 1H). The ^13^C NMR spectrum revealed 30 carbon signals, including those corresponding to a carboxyl group (δ 178.78, C-28) and a trisubstituted double bond (δ 124.99, C-12; 138.74, C-13). These spectral features, in comparison with literature data (Aguirre et al., 2006), led to the identification of compound 1 as asiatic acid.

Compound 2 displayed a molecular ion peak at *m/z* 471.3481 [M − H]^−^, corresponding to the formula C_30_H_48_O_4_. Its ^1^H NMR spectrum featured seven methyl group signals [*δ* 1.10 (s, 3H), 0.93 (s, 3H), 0.91 (s, 3H), 0.88 (s, 6H), 0.71 (s, 6H)] and a carboxylic acid proton (*δ* 12.04, s, 1H). The ^13^C NMR spectrum showed 30 carbon signals, including a carboxyl carbon (δ 179.06, C-28) and two olefinic carbons (δ 121.90, C-12; 144.38, C-13), consistent with the structure of maslinic acid (Taniguchi et al., 2002).

Compound 3 showed a molecular ion peak at *m/z* 455.3531 [M − H]^−^, corresponding to C_30_H_48_O_3_. The ^1^H NMR spectrum exhibited signals for seven methyl groups [δ 1.05 (s, 3H), 0.91 (s, 3H), 0.91 (s, 3H), 0.87 (s, 3H), 0.82 (d, *J* = 6.4 Hz, 3H), 0.76 (s, 3H), 0.68 (s, 3H)], an olefinic proton (δ 5.13, t, *J* = 3.6 Hz, 1H), and a carboxylic acid proton (δ 11.90, s, 1H). The ^13^C NMR data confirmed the presence of 30 carbon atoms, including a carboxyl carbon (δ 178.76, C-28), two olefinic carbons (δ 125.04, C-12; 138.66, C-13), and a hydroxylated methine carbon (δ 77.30, C-3). These data were consistent with those of ursolic acid (Seebacher et al., 2003).

Detailed spectroscopic data, including HRMS, ^1^H, and ^13^C NMR spectra for compounds 1–3, are provided in the Supplementary Data.

### 3.2 Antibacterial activity of AA, MA, and UA

Following the isolation and identification of AA, MA, and UA from ECPL, we evaluated their antibacterial activity and spectrum. The MICs of the compounds were determined against MRSA-4, *S. aureus* ATCC25923, *S. epidermidis*, and *E. faecalis* (Gram-positive), as well as *P. aeruginosa* PA14, *E. coli* ATCC25922, and *S. enterica subsp. Enterica* (Gram-negative) using the standard broth microdilution method in accordance with CLSI guidelines (Humphries et al., 2021).

As summarized in Table 1, AA, MA, and UA exhibited antibacterial activity against MRSA-4 and other tested Gram-positive bacteria. The MIC values for Gram-positive bacteria ranged from 2 to 32 μg/mL. AA and MA both exhibited MICs of 32 μg/mL against MRSA-4. UA also showed inhibitory activity against MRSA-4, with a MIC of 8 μg/mL. All three compounds demonstrated varying degrees of activity against other Gram-positive strains, with MICs falling within the same range. In contrast, the compounds demonstrated limited activity against Gram-negative bacteria, with MICs consistently exceeding 128 μg/mL for all tested strains.

**Table 1:** MIC of AA, MA and UA against bacteria (μg/mL).

Strains	AA	MA	UA	Van^a^	Oxa^b^
*S. aureus* ATCC25923	32	32	16	2	0.125
MRSA-4	32	32	8	2	64
*S. epidermidis* (1.143)	16	8	8	2	0.25
*E. faecalis*	16	4	2	1	8
*P. aeruginosa* PA14	>128	>128	>128	>16	>64
*E. coli* ATCC25922	>128	>128	>128	>16	>64
*S. enterica subsp. Enterica* (1.1114)	>128	>128	>128	>16	>64

a Van: Vancomycin.

b Oxa: Oxacillin.

### 3.3 Time-kill kinetics of AA, MA, and UA against MRSA

**Figure 1:**
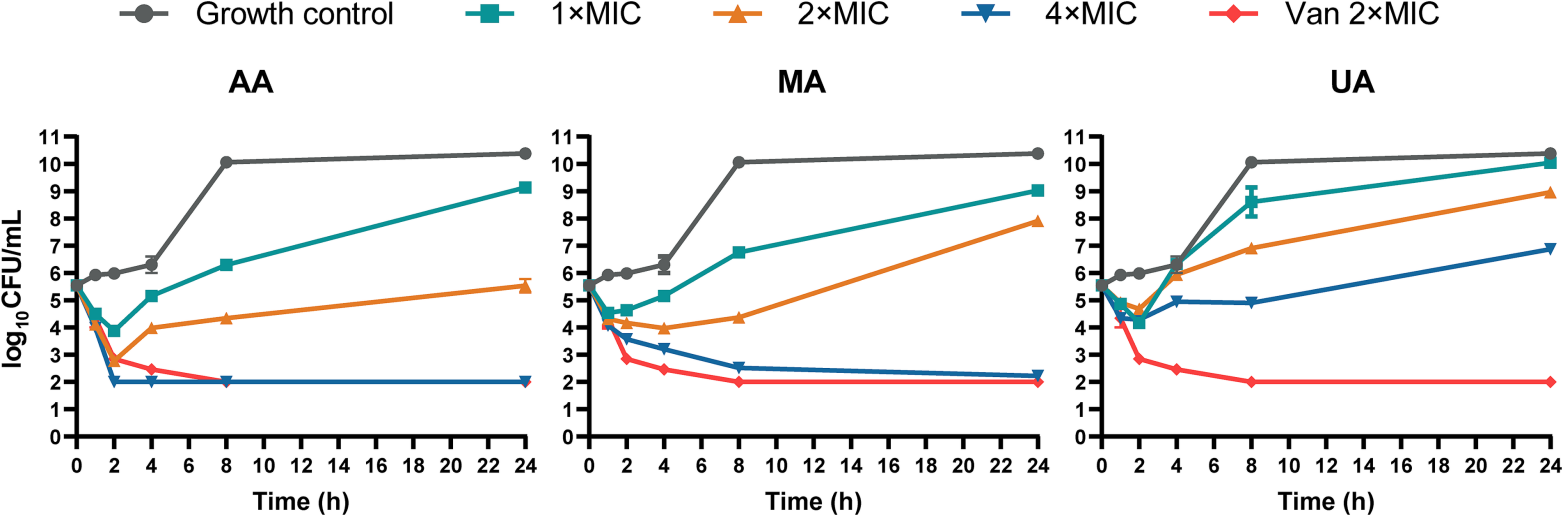
Time-kill curves of AA, MA and UA against MRSA-4. Time-kill curves showing the reduction of viable bacterial counts (MRSA-4) over 24 h upon treatment with AA, MA, and UA at indicated concentrations (1×, 2×, and 4 × MIC), vancomycin (Van) at 2 × MIC (positive control), and growth control. Data represent mean ± standard deviation (SD) from three independent biological replicates.

We next performed time-kill kinetics assays to characterize the time-dependent antibacterial effects of AA, MA, and UA against MRSA-4. Bacterial cultures were exposed to AA, MA, and UA at 1×, 2×, and 4 × their respective MICs, and viable CFU were counted at designated time points (0, 1, 2, 4, 8, and 24 h). As shown in the time-kill curves (Figure 1), treatment with 1 × or 2 × MIC of AA, MA, or UA led to an initial reduction in bacterial load within the first 2 h. However, bacterial regrowth occurred by 24 h, indicating primarily bacteriostatic activity at 1 × and 2 × MIC. In contrast, at 4 × MIC, the compounds exhibited enhanced bactericidal effects. AA showed rapid and potent bactericidal activity, achieving a > 3 log_10_ CFU/mL reduction within 2 h and reducing bacterial counts below the detection limit (<2 log_10_ CFU/mL). MA also exhibited bactericidal activity at 4 × MIC, but with slower kinetics, achieving a > 3 log_10_ CFU/mL reduction only at 24 h. UA displayed limited bactericidal activity even at 4 × MIC, failing to achieve a > 3 log_10_ reduction over 24 h, and bacterial regrowth was evident after 8 h. As a positive control, vancomycin at 2 × MIC exhibited potent bactericidal activity, reducing bacterial counts below the detection limit within 8 h.

### 3.4 Morphological alterations and elongation of *S. aureus* and MRSA induced by AA, MA, and UA treatment

**Figure 2:**
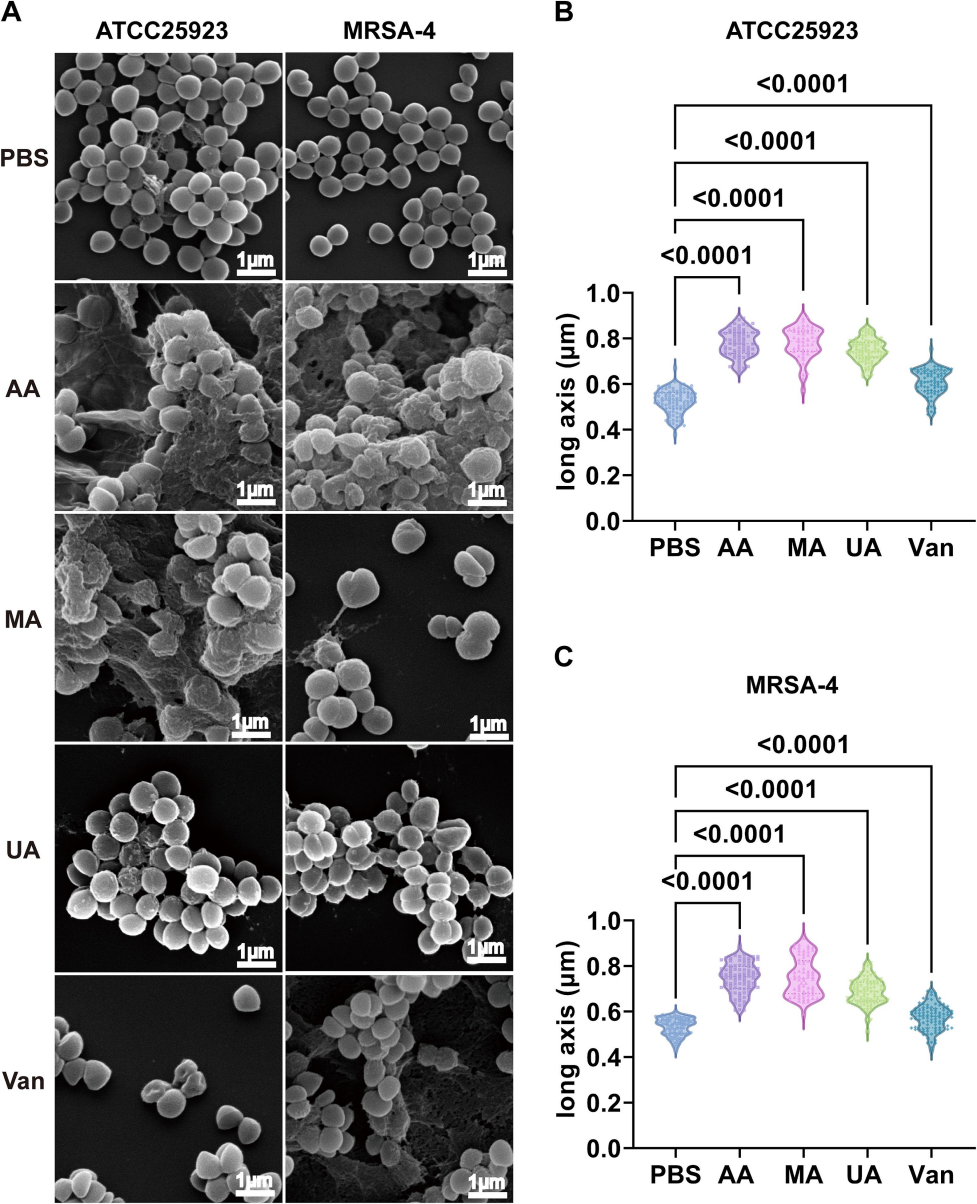
Disruption of morphology and elongation of *S. aureus* ATCC25923 and MRSA-4 cells after treatment with AA, MA, and UA. **(A)** SEM images of *S. aureus* ATCC25923 and MRSA-4 treated with PBS (control), AA, MA, UA, or vancomycin (Van). Bacteria at mid-logarithmic phase were incubated with compounds at 2 × MIC for 2 h. **(B)** Quantitative analysis of the long axis length of *S. aureus* ATCC25923 after different treatments. **(C)** Quantitative analysis of the long axis length of MRSA-4 after different treatments. Data are presented as mean ± SD and were analyzed using one-way ANOVA followed by Tukey’s multiple comparisons test.

Having established the antibacterial activity of AA, MA, and UA, we next examined their effects on bacterial morphology. We employed SEM to examine the alterations induced by these compounds in *S. aureus* ATCC25923 and MRSA-4. Bacterial cultures were treated with AA, MA, or UA at 2 × MIC for 2 h, while untreated (PBS group) cultures served as negative controls. SEM imaging revealed marked morphological changes following treatment (Figure 2A). In PBS groups, both *S. aureus* ATCC25923 and MRSA-4 maintained typical spherical morphology with smooth and intact surfaces. In contrast, bacteria treated with AA, MA, or UA exhibited substantial structural disruptions, including surface roughening, irregular shapes, and membrane damage.

Beyond these general morphological alterations, quantitative analysis of cell long-axis revealed a striking phenotype: significant cell elongation induced by the compounds (Figures 2B,C and Supplementary Table S1) revealed that AA treatment significantly increased the average long axis of *S. aureus* ATCC25923 cells from 0.52 ± 0.05 μm (control) to 0.78 ± 0.06 μm. Similar elongation phenotypes were observed in MRSA-4 cells upon treatment with AA, MA, or UA. In contrast, vancomycin-treated cells exhibited distorted shapes and membrane damage, consistent with its mechanism of action on cell wall synthesis, but did not show significant cell elongation compared to the control. These distinct morphological alterations indicate that AA, MA, and UA disrupt bacterial processes that are mechanistically different from those affected by vancomycin. Notably, the observed bacterial cell elongation, specifically the increase in cell length without proper septation, is a characteristic morphological alteration frequently associated with the inhibition of bacterial cell division (Hurley et al., 2016). Taken together, these SEM findings indicate that AA, MA, and UA exert their antibacterial effects by interfering septation with bacterial cell division.

### 3.5 AA, MA, and UA disrupt bacterial cell division by targeting septum formation, Z-ring assembly and FtsZ polymerization

Building on our findings that AA, MA, and UA induce morphological alterations indicative of cell division defects, and our previous work suggesting that ECPL impairs FtsZ dynamics, we hypothesized that these specific constituents exert their antibacterial effects by targeting the bacterial cell division protein FtsZ. To test this hypothesis, we conducted a stepwise series of experiments investigating their impact on septum formation, Z-ring assembly, and FtsZ polymerization.

**Figure 3:**
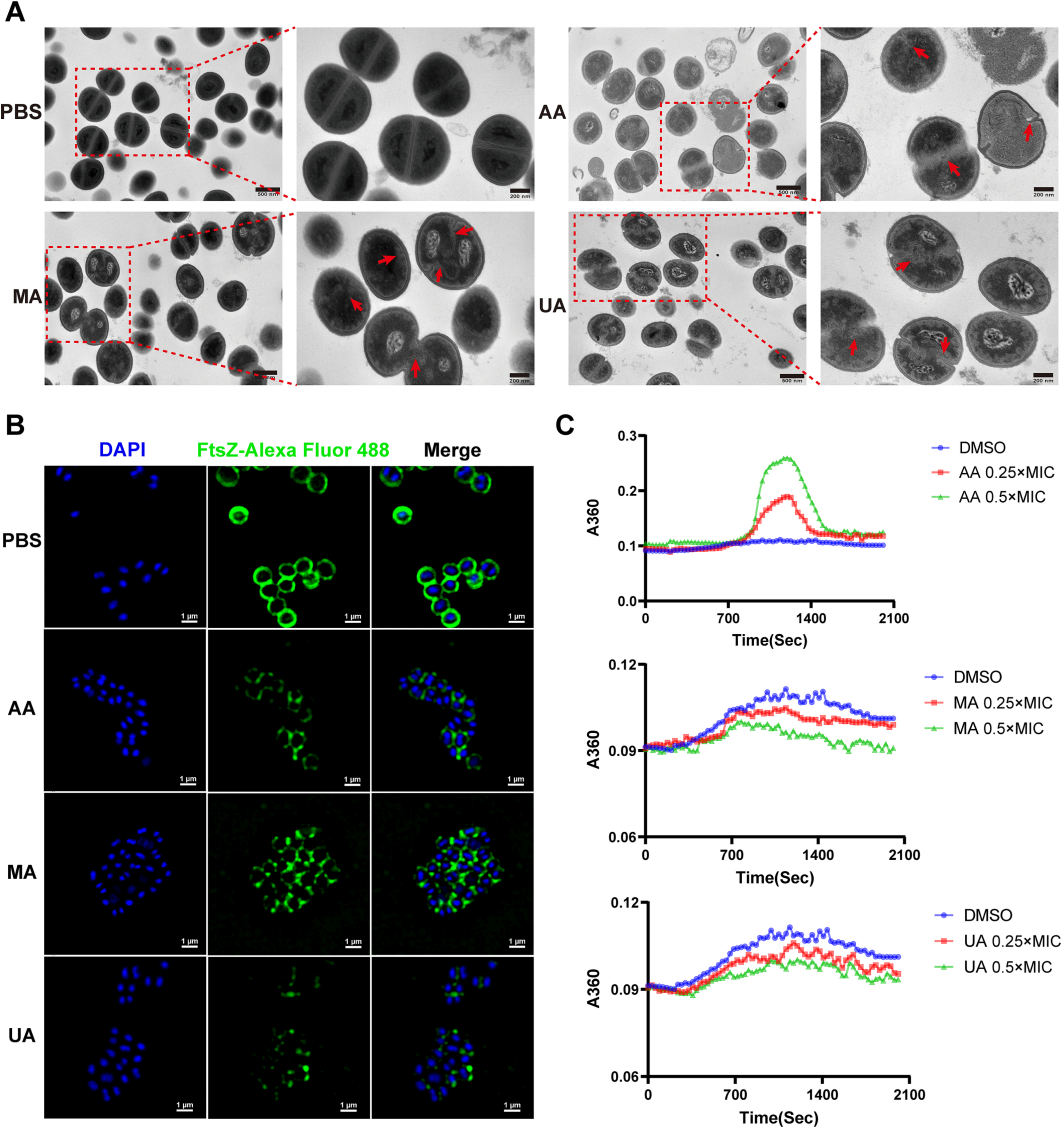
AA, MA, and UA disrupt bacterial cell division by targeting septum formation, Z-ring assembly and FtsZ polymerization. **(A)** TEM images illustrating the impact of AA, MA, and UA on septum formation and cell division in MRSA-4. Bacteria were treated with compounds at 1 × MIC for 4 h. The red arrows highlight enlarged cells resulting from AA, MA, or UA treatment, where aberrant septal structures are mislocalized. Scale bar = 200 nm. **(B)** Representative SIM images of fixed MRSA-4 cells, immunostained for FtsZ and DAPI-stained for nucleoid, following treatment with or without 1 × MIC AA, MA, and UA for 4 h. Scale bar = 1 μm. **(C)** Concentration-dependent effects of AA, MA, and UA on SaFtsZ polymerization, assessed by monitoring time-dependent changes in absorbance at A360 at 37°C. Polymerization profiles were recorded in the presence of DMSO as a vehicle or at the indicated concentrations of AA, MA, and UA.

We first evaluated the impact of AA, MA, and UA on septum formation in MRSA-4 cells using TEM. MRSA-4 cultures were treated with AA, MA, or UA at 1 × MIC for 4 h, with PBS-treated cells serving as the control. TEM revealed substantial defects in septum formation following compound treatment (Figure 3A, red arrows). Untreated cells displayed normal ultrastructure with well-defined, mid-cell septa, indicative of regular division. In contrast, cells treated with AA, MA, or UA often exhibited incomplete, mislocalized, or absent septa, indicating impaired cytokinesis. These abnormalities imply that AA, MA, and UA disrupt the septum formation process, which is essential for successful cell division.

Given that septum formation is orchestrated by the FtsZ-based Z-ring, we next examined whether AA, MA, and UA affect Z-ring assembly. Structured illumination microscopy (SIM) was employed to visualize FtsZ localization in MRSA-4 cells treated with each compound at 1 × MIC for 4 h. Immunofluorescence staining revealed that in untreated cells, FtsZ formed distinct, mid-cell Z-rings, characteristic of actively dividing bacteria. In contrast, compound-treated cells exhibited aberrant FtsZ localization, including fragmented, incomplete, or diffuse cytoplasmic patterns, indicating defective Z-ring formation (Figure 3B). These findings suggest that AA, MA, and UA interfere with the proper spatial organization and assembly of the Z-ring.

To determine whether the disruptions of Z-ring were due to direct effects on FtsZ polymerization, we performed *in vitro* polymerization assays. FtsZ polymerization was assessed by monitoring the time-dependent increase in A_360_ upon induction using a microplate reader. Briefly, 10 μM SaFtsZ was pre-incubated with AA, MA, UA or DMSO, and polymerization was initiated by adding K^+^, Mg^2+^, and GTP. As shown in Figure 3C, AA significantly enhanced FtsZ polymerization, indicated by an elevated A_360_ values compared to the DMSO control. In contrast, both MA and UA markedly inhibited FtsZ polymerization, resulting in decreased A_360_ values. These results demonstrate that AA, MA, and UA directly modulate FtsZ polymerization dynamics in *vitro*, providing a molecular explanation for their disruption of Z-ring assembly.

Taken together, these findings from ultrastructural analysis (TEM), subcellular localization (SIM), and biochemical assays provide compelling evidence that AA, MA, and UA impair bacterial division by targeting FtsZ dynamics.

### 3.6 Binding affinity and interaction modes of AA, MA, and UA with FtsZ

Having established that AA, MA, and UA impair bacterial cell division by disrupting FtsZ dynamics, we next explored the molecular interactions between these compounds and FtsZ to understand the structural basis of their binding and distinct functional effects.

**Figure 4:**
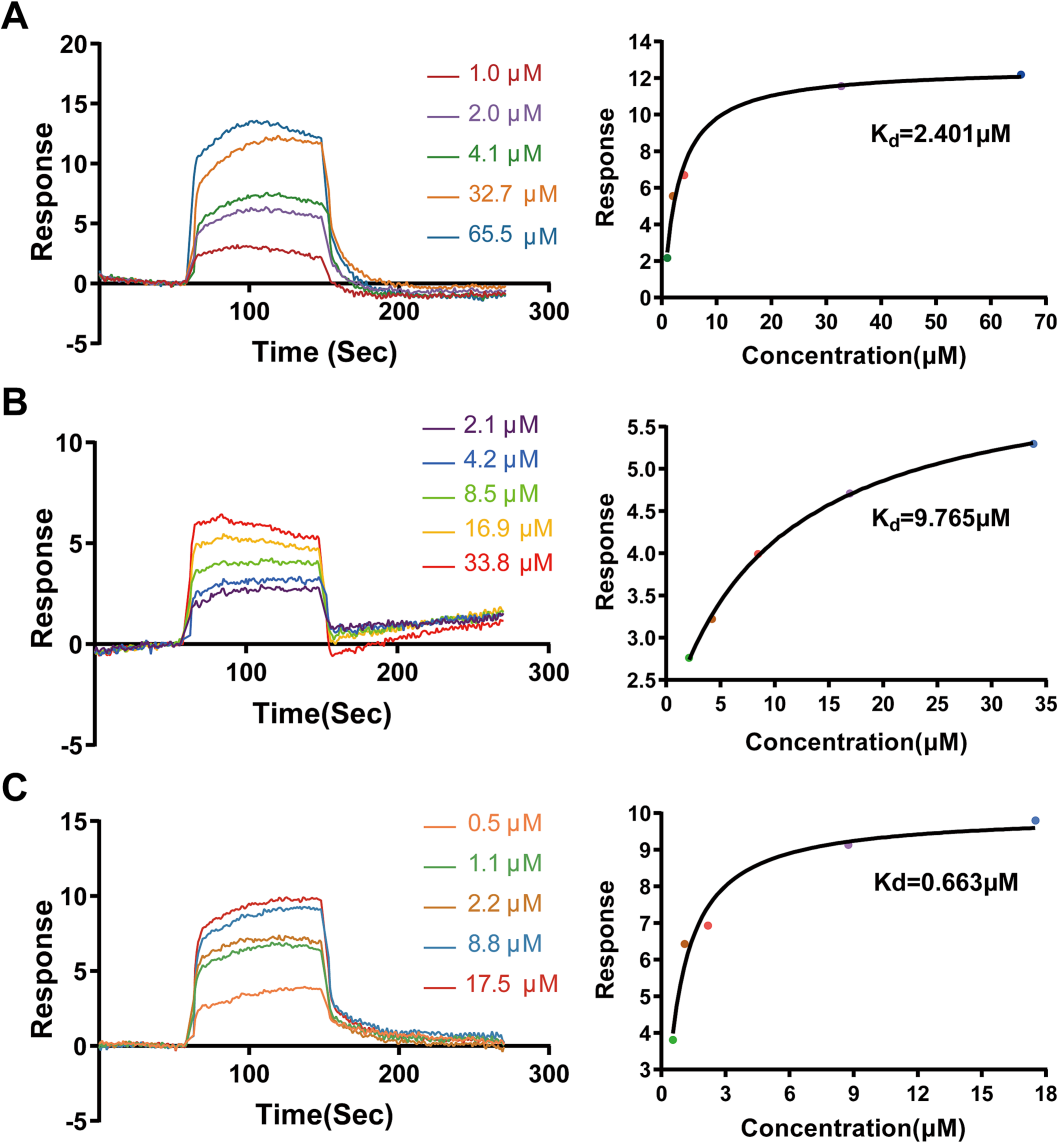
Binding analysis of AA, MA, and UA to SaFtsZ by SPR. SPR sensorgrams showing the binding of AA **(A)**, MA **(B)**, and UA **(C)** to immobilized SaFtsZ. The left panels depict real-time binding responses (RU) upon injection of increasing analyte concentrations. The right panels show steady-state binding plots, with Kd values calculated using a binding model.

We first performed SPR spectroscopy to experimentally measure the binding affinities of AA, MA, and UA for immobilized SaFtsZ protein. The SPR sensorgrams demonstrated concentration-dependent binding (Figures 4A–C), yielding equilibrium dissociation constants (Kd) of 2.401 μM (AA), 9.765 μM (MA), and 0.663 μM (UA). These results experimentally confirming their direct interaction with FtsZ.

**Figure 5:**
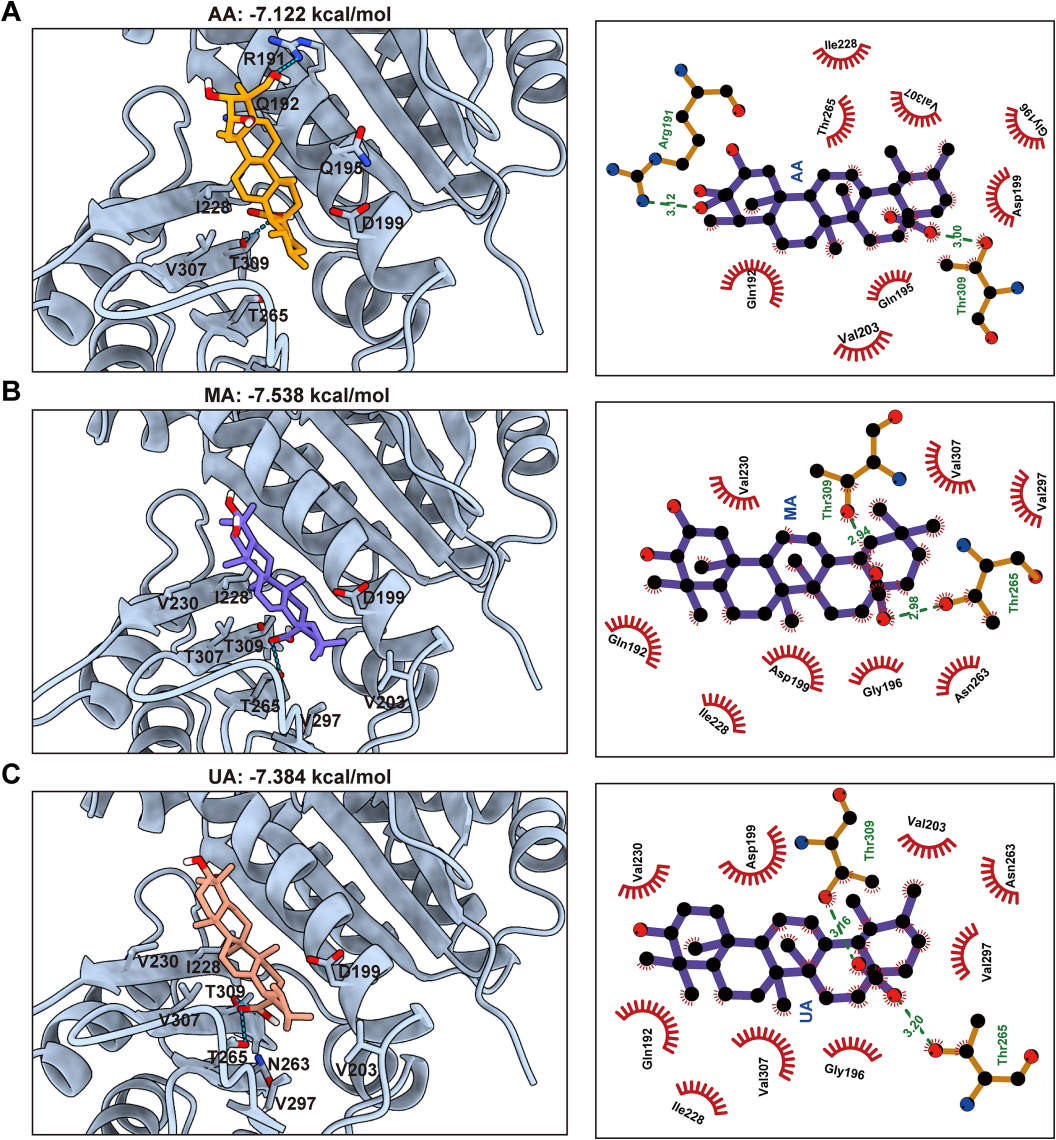
Predicted binding modes and interactions of AA, MA, and UA with FtsZ. Molecular docking predictions of AA **(A)**, MA **(B)**, and UA **(C)** binding to FtsZ (PDB ID: 6KVP), with docking scores indicated. Left panels: Predicted 3D binding poses showing compounds (colored sticks) within the SaFtsZ pocket (light blue cartoon), with key interacting residues labeled. Hydrogen bonds are shown as green dashed lines. Right panels: 2D interaction diagrams illustrating hydrogen bonds (green dashed lines, with distances in Å) and hydrophobic contacts (red arcs). Key interacting residues are labeled.

To complement these experimental findings and gain structural insights into the binding modes, molecular docking studies were conducted using the FtsZ crystal structure (PDB ID: 6KVP) and AutoDock Vina. The docking results consistently predicted that AA, MA, and UA bind to a common hydrophobic inter-domain cleft, which is formed by the H7 helix, T7 loop, and adjacent *β*-sheets (Supplementary Figures 1A,B). The calculated docking scores were −7.12 kcal/mol for AA, −7.54 kcal/mol for MA, and −7.38 kcal/mol for UA (Figures 5A–C). These docking scores align well with the experimental binding affinities determined by SPR, thereby providing computational validation for the observed binding. Specifically, AA is predicted to form hydrogen bonds with Arg191 in the H7 helix and with Thr309 in the C-terminal subdomain via its hydroxyl and carboxyl groups, respectively. In a similar manner, both MA and UA are predicted to form hydrogen bonds with Thr265 and Thr309 in the C-terminal subdomain through their carboxyl groups. In addition to these polar interactions, multiple hydrophobic contacts were predicted between AA, MA, and UA, and surrounding amino acid residues at the binding site, including Gln192, Gln195, Gly196, Asp199, Val203, Ile228, and Val307. These combined hydrogen bonding and hydrophobic interactions provide a structural basis for the observed binding affinities measured by SPR and offer insight into how these compounds may disrupt FtsZ function by binding to this site.

### 3.7 AA exhibits a distinct docking conformation compared to MA and UA

Building on the molecular docking results that predicted a common binding site for AA, MA, and UA on FtsZ, we next conducted a comparative analysis of their predicted binding conformations and specific interactions to understand how structural differences among the ligands influence their binding modes and potentially their distinct effects on FtsZ function.

**Figure 6:**
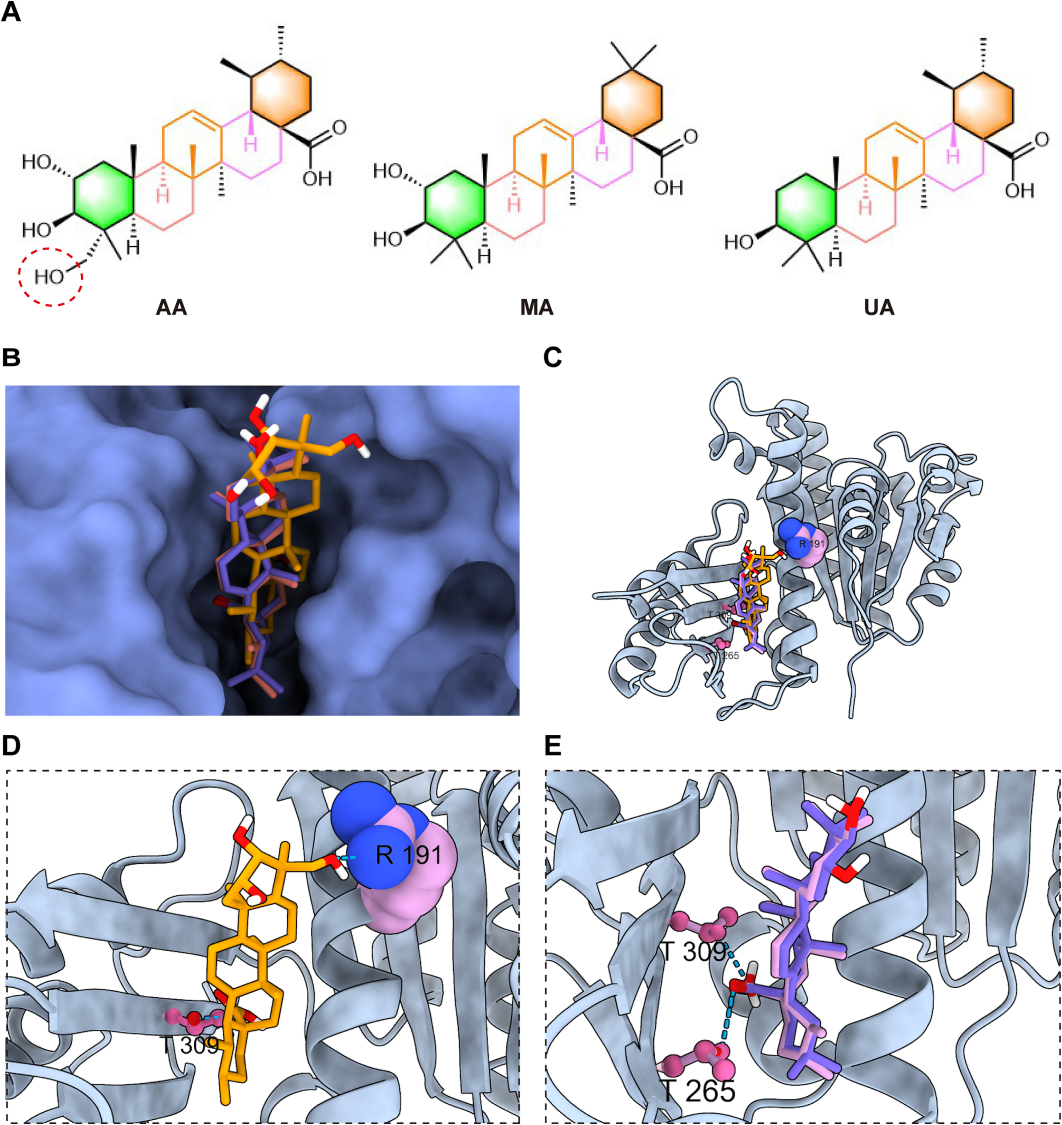
AA exhibits a distinct docking conformation compared to MA and UA. **(A)** Chemical structures of AA, MA, and UA. **(B,C)** Superimposed docking models of AA, MA, and UA bound to FtsZ, shown as surface and cartoon representations. **(D)** Close-up view of AA’s interaction with the binding site of FtsZ. **(E)** Close-up view of the interactions of MA and UA with the poket of FtsZ, displayed together. Amino acid residues are shown in ball-stick or sphere representation, with hydrogen bonds indicated by cyan dashed lines.

A key structural difference among the three compounds is the presence of an additional hydroxyl group in AA compared to MA and UA (Figure 6A). Detailed analysis and superimposition of the predicted docking models revealed that although all three compounds bind to the same FtsZ pocket, AA adopts a distinct binding conformation and orientation within the site compared to MA and UA (Figures 6B,C). Specifically, AA’s conformation is notably shifted relative to the poses of MA and UA. Further examination of the predicted interactions provides insights into the reasons for this conformational difference (Figures 6D,E). As discussed previously, all three compounds form hydrogen bonds with residues in the C-terminal subdomain. However, the unique hydroxyl group of AA was predicted to form an additional hydrogen bond with Arg191 on the H7 helix, an interaction not observed for MA or UA. This specific interaction between AA’s hydroxyl group and Arg191 appears to be a key determinant of AA’s distinct spatial orientation within the binding site. While MA and UA also engage in hydrogen bonding, their interaction patterns are distinct from AA, for instance, forming hydrogen bonds with Thr265 in the C-terminal subdomain. These differences in the specific ligand-protein interactions and resulting binding conformations of AA, MA, and UA within the FtsZ binding pocket likely induce distinct local conformational changes in FtsZ, which in turn could differentially affect its dynamic functions, such as polymerization. This hypothesis is consistent with and provides a structural basis for the varying effects observed in our previous *in vitro* FtsZ polymerization assays, where AA promoted polymerization while MA and UA inhibited it.

### 3.8 AA is effective in the murine skin infection model

**Figure 7:**
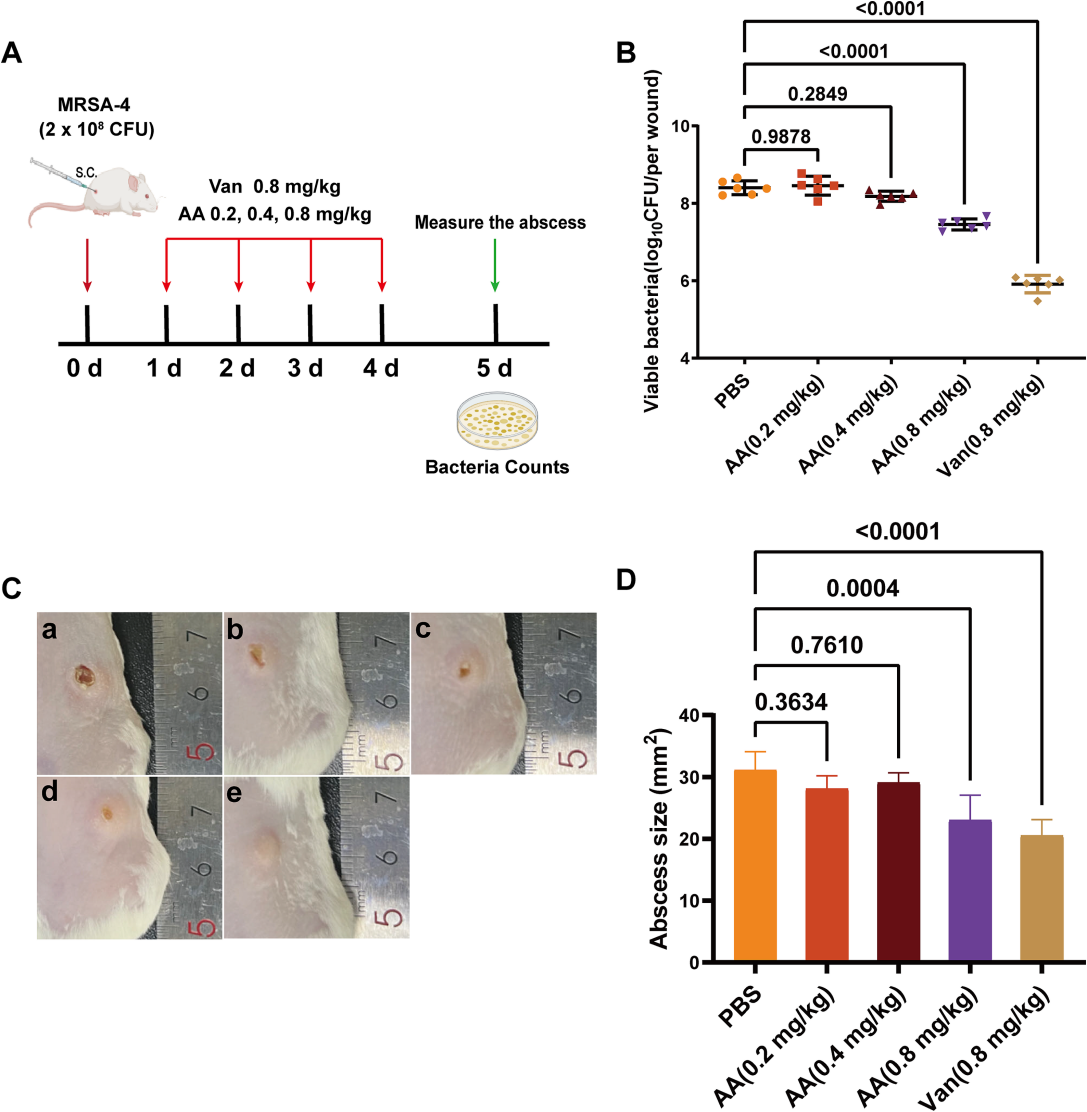
*In vivo* antibacterial efficacy of AA in a murine MRSA-4 skin infection model. **(A)** Schematic diagram illustrating the experimental design for evaluating the *in vivo* antibacterial activity of AA in a MRSA-4 skin infection mouse model. **(B)** Quantification of bacterial loads (MRSA-4) in skin wounds after AA treatment, assessed by CFU counts on day 5 post-infection (*n* = 6). **(C)** Representative images of infected skin wounds at 5 days post-infection under different treatment conditions: a: PBS (negative control); b: AA at 0.2 mg/kg; c: AA at 0.4 mg/kg; d: AA at 0.8 mg/kg; e: vancomycin at 0.8 mg/kg (positive control). **(D)** Measurement of wound sizes in different treatment groups at 5 days post-infection. Data are presented as mean ± SD. Statistical significance was determined by one-way ANOVA followed by Tukey’s multiple comparisons test.

Building on its rapid bactericidal activity *in vitro* and unique FtsZ-binding profile, we next evaluated the *in vivo* therapeutic efficacy of AA using a murine skin infection model. The model was established in BALB/c mice by subcutaneous inoculation with 2 × 10^8^ CFU of MRSA-4 (Figure 7A). Treatment began 24 h post-infection and consisted of daily subcutaneous injections at the infection site for four consecutive days with AA (0.2, 0.4, or 0.8 mg/kg), vancomycin (at 0.8 mg/kg; positive control), or PBS (negative control). Therapeutic outcomes were assessed on day 5 post-infection by quantifying bacterial burden, visually monitoring wound healing, and measuring abscess size.

Quantification of bacterial loads in infected skin tissues (Figure 7B) revealed a dose-dependent reduction in subcutaneous MRSA burden with increasing AA concentrations. Specifically, treatment with AA resulted in varying degrees of reduction in bacterial load compared to the PBS group. Low-dose AA (0.2 and 0.4 mg/kg) yielded modest but dose-responsive decreases in bacterial load, while 0.8 mg/kg AA significantly reduced bacterial counts compared to the PBS group. Notably, the antibacterial efficacy of 0.8 mg/kg AA was comparable to that of vancomycin at the same dose.

Visual inspection of wound healing throughout the treatment period (Figure 7C) demonstrated a dose-dependent improvement in lesion appearance with AA treatment. PBS-treated mice displayed severe infection symptoms, including extensive necrosis and abscess formation, with no visible healing. Mice treated with low-dose AA exhibited moderate, dose-dependent improvements in wound appearance. In contrast, 0.8 mg/kg AA resulted in substantial amelioration of symptoms, including reduced redness, swelling, and lesion size—comparable to outcomes observed in vancomycin-treated mice. Quantitative analysis of abscess size (Figure 7D) further supported these findings: 0.8 mg/kg AA significantly reduced abscess dimensions relative to PBS, with an effect size similar to vancomycin.

Together, these *in vivo* results demonstrate that AA effectively treats subcutaneous MRSA skin infections in mice, significantly reducing bacterial burden, promoting wound healing, and suppressing abscess formation.

## 4 Discussion

**Figure 8:**
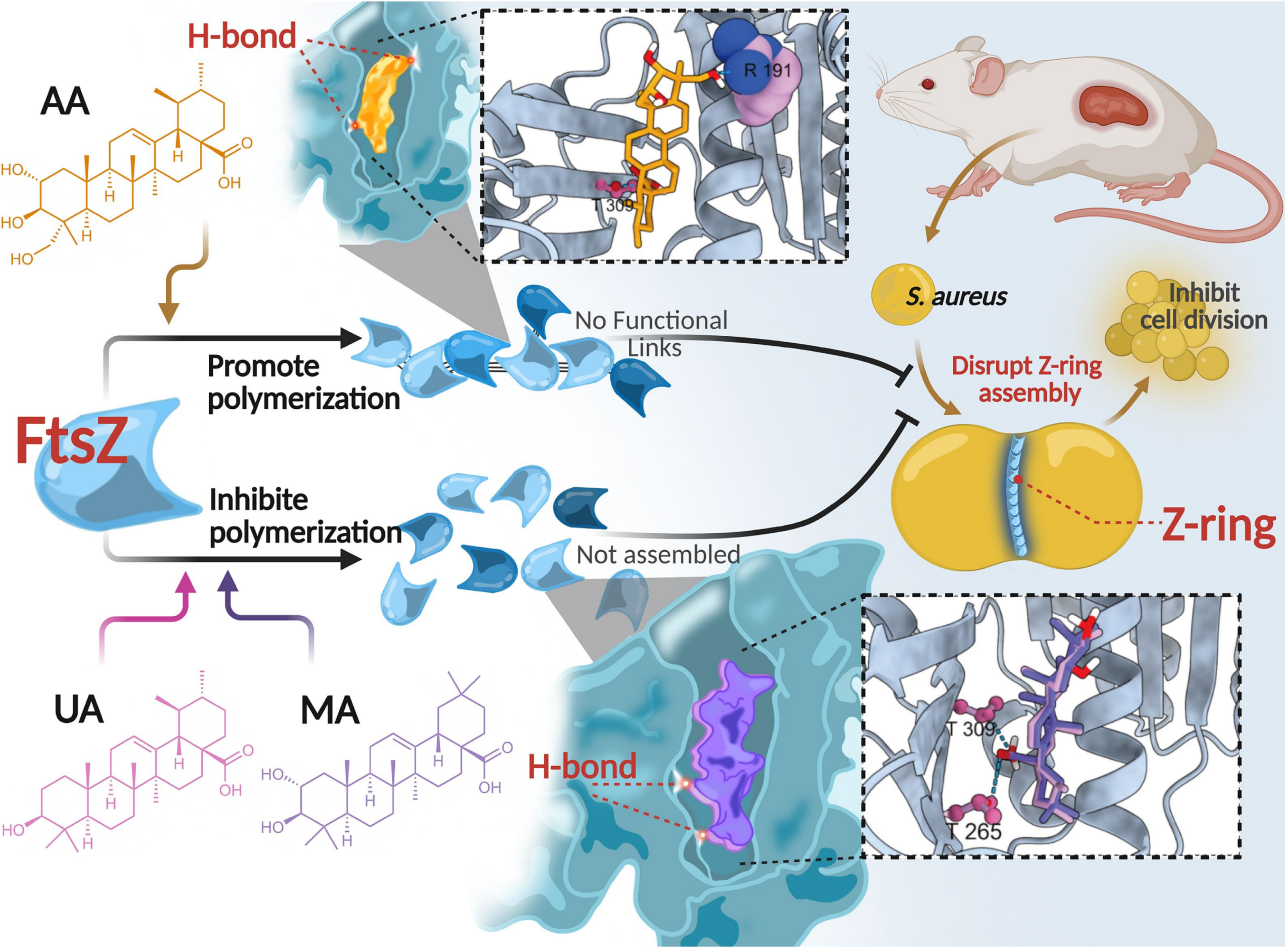
Proposed mechanism of action of AA, MA, and UA. AA, MA, and UA target FtsZ, differentially modulating its polymerization dynamics and disrupting Z-ring assembly, thereby inhibiting cell division.

In this study, we identified and characterized three compounds—AA, MA, and UA—isolated from *Cyclocarya paliurus* leaves as novel anti-MRSA agents that act by directly targeting FtsZ. Our comprehensive investigation delineated their distinct modes of FtsZ modulation. Specifically, our results demonstrate that AA, MA, and UA interact with FtsZ, differentially modulate its polymerization dynamics, impair Z-ring assembly, and ultimately block bacterial cell division (Figure 8). Among these compounds, AA emerged as a particularly promising lead candidate, exhibiting potent bactericidal activity and robust *in vivo* efficacy.

These compounds have been reported to exhibit antibacterial activity against various pathogens, including *S. aureus* (Liu et al., 2015; Cargnin and Gnoatto, 2017; Sycz et al., 2022). Previous studies suggested that their antimicrobial effects might be associated with alterations in bacterial membrane integrity, gene expression, and biofilm formation (Sycz et al., 2022); however, the specific molecular targets have remained undefined. In this study, we provide compelling evidence that FtsZ is a direct intracellular target of AA, MA, and UA. Treatment of *S. aureus* and MRSA with these compounds induced a characteristic cell elongation phenotype—a morphological hallmark of defective cell division (Hurley et al., 2016). SPR assays confirmed direct binding of all three compounds to FtsZ, with Kd values in the low micromolar range (AA: 2.401 μM; MA: 9.765 μM; UA: 0.663 μM), establishing a direct molecular interaction between these compounds and FtsZ.

Intriguingly, we observed a notable divergence between the *in vitro* FtsZ binding affinities, MICs, and bactericidal kinetics of the three compounds. For instance, despite possessing the highest binding affinity (Kd = 0.663 μM) and lowest MIC against MRSA (8 μg/mL), UA acted primarily bacteriostatically in time-kill assays. Conversely, AA, with a lower affinity (Kd = 2.401 μM) and a higher MIC (32 μg/mL), exhibited the most rapid bactericidal profile among the three compounds. MA also showed bactericidal activity, albeit with slower kinetics. This disparity highlights that target binding affinity or simple MIC values may not fully predict the speed or nature of the antibacterial effect, particularly the crucial bactericidal potency needed to combat resistant infections.

This divergence in antibacterial outcomes likely stems from differential effects on FtsZ dynamics, driven by distinct binding interactions within the common binding pocket. *In vitro* FtsZ polymerization assays revealed strikingly divergent effects on FtsZ assembly: AA promoted FtsZ polymerization, whereas both MA and UA inhibited it. Molecular docking studies provided a structural basis for these functional distinctions. While all three compounds bind to a shared hydrophobic inter-domain cleft on FtsZ, AA adopts a distinct conformation within this pocket, mediated by unique interactions such as a hydrogen bond with Arg191 located on the H7 helix, an interaction not observed for MA or UA. In contrast, MA and UA were predicted to primarily form hydrogen bonds with residues including Thr265 and Thr309, which contribute to shaping the ligand-binding pocket and influencing binding affinity and resistance (Fujita et al., 2017; Ferrer- González et al., 2019; Huecas et al., 2021; Du et al., 2022). The H7 helix itself is critical for FtsZ conformational stability and can impact polymerization dynamics (Elsen et al., 2012). Therefore, these distinct interaction patterns within a common binding region likely induce differential conformational changes in FtsZ, resulting in the observed opposing effects on polymerization dynamics (promotion vs. inhibition). AA’s promotion of FtsZ polymerization may lead to the formation of overly stable or non-productive polymers, thereby disrupting the dynamic equilibrium essential for effective Z-ring constriction and rapid cell death. Similar FtsZ-targeting agents that modulate polymerization have been reported (Margalit et al., 2004; Kaul et al., 2012; Sun et al., 2017). Conversely, the inhibition observed for MA and UA is consistent with the mechanism of known FtsZ inhibitors that disrupt the balance between assembly and disassembly (Plaza et al., 2010; Bhondwe et al., 2024).

Despite these divergent effects on FtsZ polymerization and distinct binding modes, all compounds ultimately disrupted Z-ring assembly and septum formation at the cellular level. As shown by cellular imaging (SIM) and ultrastructural analysis (TEM), treated cells exhibited severe disruptions in Z-ring localization and assembly, leading to substantial defects in septum formation, including incomplete or absent septa. These findings corroborate that regardless of their specific mode of FtsZ modulation, these compounds effectively interfere with the bacterial divisome.

Translating these *in vitro* and mechanistic findings to a therapeutic setting, the *in vivo* efficacy of AA was validated in a murine subcutaneous MRSA skin infection model. AA significantly reduced the bacterial burden and accelerated wound healing compared to the PBS control, with efficacy comparable to vancomycin. These results highlight the therapeutic promise of AA as a lead candidate for treating MRSA infections.

Nevertheless, our study has several limitations that merit future investigation. While molecular docking provided valuable predictions regarding binding interactions and conformations, experimental validation—such as site-directed mutagenesis, or X-ray crystallography—would strengthen the mechanistic insights and confirm the proposed binding modes. In addition, a more detailed characterization of FtsZ polymerization and depolymerization kinetics in the presence of these compounds could offer deeper understanding of their modulatory effects. Molecular dynamics simulations may further help clarify the link between specific interactions and their impact on FtsZ dynamics. Additionally, the *in vivo* efficacy of AA was demonstrated in a localized skin infection model. To further advance its therapeutic potential, future studies involving systemic infection models, as well as comprehensive pharmacokinetic/pharmacodynamic profiling and resistance evaluation, would be valuable.

In conclusion, we identified AA, MA, and UA as a novel class of natural antibacterial agents that may disrupt bacterial cell division by targeting septum formation and the Z-ring assembly. Despite binding to a common site, these compounds modulate FtsZ dynamics in distinct ways, ultimately impairing Z-ring assembly. Notably, AA demonstrated potent and rapid bactericidal activity both *in vitro* and *in vivo*, supporting its potential as a promising lead compound for the development of new anti-MRSA therapies.

## Data Availability

The original contributions presented in the study are included in the article/Supplementary material, further inquiries can be directed to the corresponding authors.
