# Supplementary material for: Compounds from Cyclocarya paliurus leaves inhibit binary division of methicillin-resistant Staphylococcus aureus by disrupting FtsZ dynamic
Source: Front Microbiol. 2025 Jun 18;16:1622623. doi: 10.3389/fmicb.2025.1622623 (PMC12213745; doi:10.3389/fmicb.2025.1622623)
Supplement: Supplementary file 1 [file Data_Sheet_1.docx]

Supplementary Material

# Identification of Compounds

Compound **1**: White power, HRMS m/z 487.3432 [M− H]^-^, molecular formula: C_30_H_48_O_5_ (calculated m/z 487.3429). ^1^H NMR (400 MHz, DMSO‑d6) δ 11.93 (s, 1H), 5.13 (d, *J* = 3.7 Hz, 1H), 4.40 (t, *J* = 5.2 Hz, 1H), 4.24 – 4.12 (m, 2H), 3.47 (d, *J* = 11.1 Hz, 1H), 3.29 (d, *J* = 4.6 Hz, 1H), 3.17 (d, *J* = 9.4 Hz, 1H), 3.04 (dd, *J* = 10.6, 4.2 Hz, 1H), 2.11 (d, *J* = 11.2 Hz, 1H), 1.95 (dd, *J* = 13.4, 4.1 Hz, 1H), 1.90 – 1.84 (m, 2H), 1.77 (dd, *J* = 12.7, 4.4 Hz, 2H), 1.61 – 1.16 (m, 13H), 1.05 (s, 3H), 0.99 (d, *J* = 13.5 Hz, 2H), 0.92 (d, *J* = 5.7 Hz, 6H), 0.82 (d, *J* = 6.3 Hz, 3H), 0.74 (s, 3H), 0.54 (s, 3H); ^13^C NMR (100 MHz, DMSO‑d6) δ 178.78, 138.74, 124.99, 76.00, 67.90, 64.37, 52.84, 47.43, 47.28, 46.45, 42.96, 42.21, 40.65, 38.96, 38.88, 37.76, 36.79, 32.64, 30.64, 27.96, 24.27, 23.79, 23.44, 21.56, 17.89, 17.51, 17.46, 17.36, 14.26. Comparison of the obtained NMR data with literature data identified compound **1** as asiatic acid.

Compound **2**: White powder, HRMS m/z 471.3481 [M - H]^-^, molecular formula: C_30_H_48_O_4_ (calculated m/z 471.3480). ^1^H NMR (400 MHz, DMSO‑d6) δ 12.04 (s, 1H), 5.20 – 5.16 (m, 1H), 4.37 (dd, *J* = 13.0, 4.6 Hz, 1H), 4.29 (d, *J* = 4.3 Hz, 1H), 3.49 – 3.40 (m, 1H), 2.80 – 2.71 (m, 2H), 1.90 (dd, *J* = 13.3, 4.0 Hz, 1H), 1.84 (dt, *J* = 6.5, 3.5 Hz, 2H), 1.75 (dd, *J* = 12.5, 4.4 Hz, 1H), 1.65 (d, *J* = 4.2 Hz, 1H), 1.62 (d, *J* = 4.1 Hz, 1H), 1.50 (d, *J* = 5.0 Hz, 1H), 1.46 (d, *J* = 4.1 Hz, 2H), 1.43 (d, *J* = 2.7 Hz, 1H), 1.34 (m, 3H), 1.24 (d, *J* = 10.5 Hz, 1H), 1.10 (s, 3H), 1.08 – 0.97 (m, 3H), 0.93 (s, 3H), 0.91 (s, 3H), 0.88 (s, 6H), 0.85 – 0.73 (m, 3H), 0.71 (s, 6H); ^13^C NMR (100 MHz, DMSO‑d6) δ 179.06, 144.38, 121.90, 82.68, 67.59, 56.49, 55.21, 47.53, 47.30, 46.14, 45.90, 41.81, 41.24, 39.40, 38.12, 33.77, 33.30, 32.77, 32.55, 30.88, 29.27, 27.62, 26.11, 23.84, 23.46, 23.04, 18.52, 17.60, 17.33, 16.78. Comparison of the obtained NMR data with literature data identified compound **2** as maslinic acid.

Compound **3**: White powder, HRMS m/z 455.3531 [M - H]^-^, molecular formula: C_30_H_48_O_3_ (calculated m/z 455.3531). ^1^H NMR (400 MHz, DMSO‑d6) δ 11.90 (s, 1H), 5.13 (t, *J* = 3.6 Hz, 1H), 4.40 – 4.27 (m, 1H), 3.49 – 3.40 (m, 1H), 2.99 (d, *J* = 8.8 Hz, 1H), 2.11 (d, *J* = 11.2 Hz, 1H), 1.99 – 1.75 (m, 4H), 1.51 (m, 11H), 1.29 (m, 4H), 1.07 (d, *J* = 7.0 Hz, 1H), 1.05 (s, 3H), 1.03 – 0.98 (m, 1H), 0.91 (s, 3H), 0.91 (s, 3H), 0.87 (s, 3H), 0.82 (d, *J* = 6.4 Hz, 3H), 0.76 (s, 3H), 0.68 (s, 3H); ^13^C NMR (100 MHz, DMSO‑d6) δ 178.76, 138.66, 125.04, 77.30, 55.24, 52.84, 47.48, 47.30, 42.11, 40.66, 38.97, 38.90, 38.85, 38.69, 37.00, 36.79, 33.17, 30.65, 28.73, 28.01, 27.46, 24.27, 23.74, 23.32, 21.55, 18.46, 17.49, 17.39, 16.56, 15.70. Comparison of the obtained NMR data with literature data identified Compound **3** as ursolic acid.

# Supplementary Table and Figure

## Supplementary Table

**Table S1**

Length of long axis of *S. aureus* ATCC25923 and MRSA-4 cells treated with AA, MA, UA, and Van (μm).

|  | ATCC25923 | MRSA-4 |
| --- | --- | --- |
| PBS | 0.52 ± 0.05 | 0.53 ± 0.04 |
| AA | 0.78 ± 0.06 | 0.75 ± 0.07 |
| MA | 0.78 ± 0.07 | 0.76 ± 0.10 |
| UA | 0.75 ± 0.05 | 0.69 ± 0.06 |
| Van | 0.61 ± 0.06 | 0.57 ± 0.06 |

## Supplementary Figure


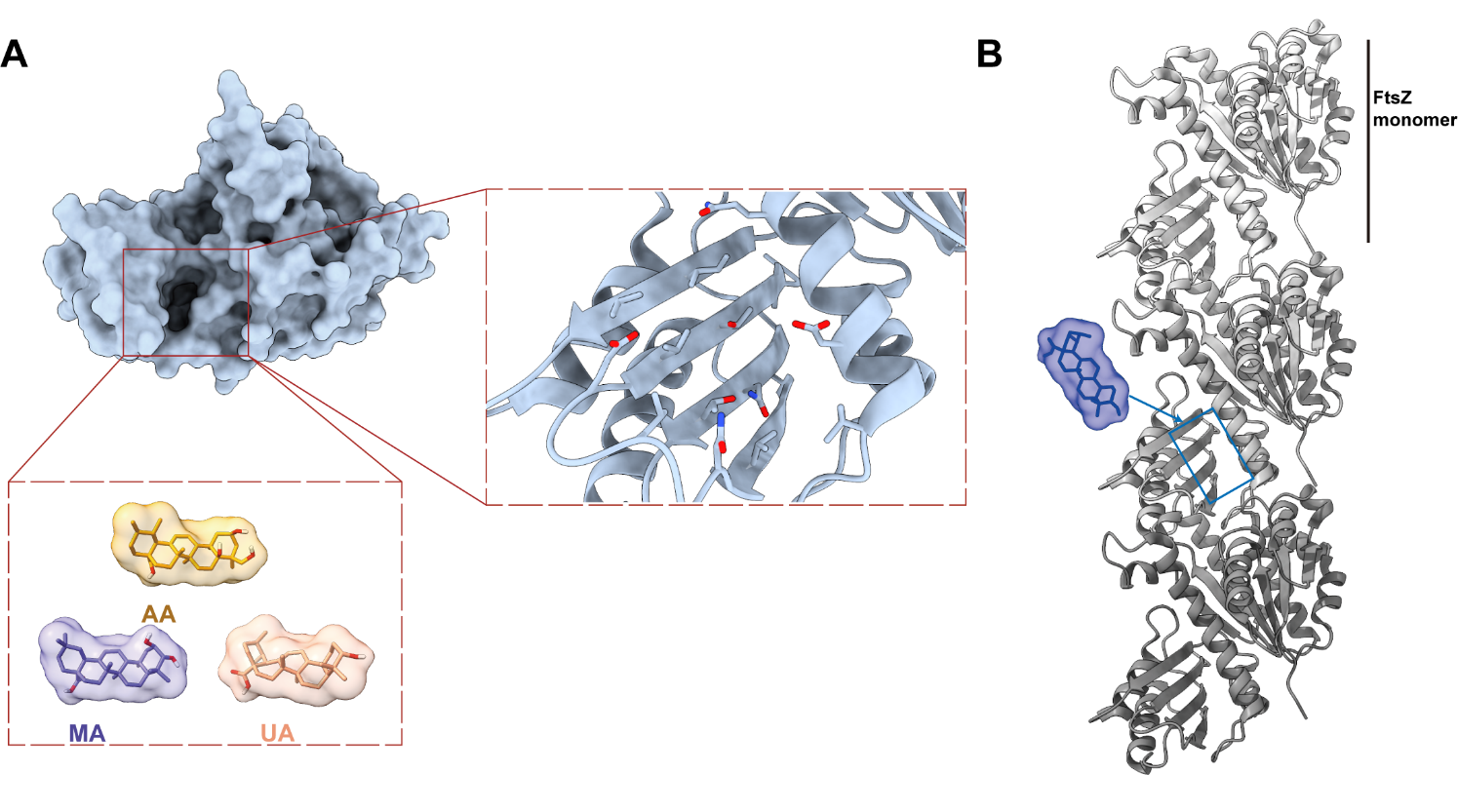


**Supplementary Figure 1.** **Structural representation of SaFtsZ and predicted compound binding site.** (**A**) Surface and cartoon representations of the predicted active sites on SaFtsZ (PDB ID: 6KVP), highlighting the interaction interfaces of AA (orange), MA (slate blue), and UA (salmon) with the protein. (**B**) Modeled structure of a S. aureus FtsZ filament composed of three monomers (shades of gray), with the compound highlighted in blue and its predicted binding site outlined with a blue box.
